# Mycobiota Composition of Robiola di Roccaverano Cheese along the Production Chain

**DOI:** 10.3390/foods10081859

**Published:** 2021-08-11

**Authors:** Federica Biolcati, Ilario Ferrocino, Maria Teresa Bottero, Alessandra Dalmasso

**Affiliations:** 1 Dipartimento di Scienze Veterinarie, Università di Torino, Largo Braccini 2, 10095 Grugliasco, TO, Italy; mariateresa.bottero@unito.it (M.T.B.); alessandra.dalmasso@unito.it (A.D.); 2 Dipartimento di Scienze Agrarie, Forestali e Alimentari (DISAFA), Università di Torino, Largo Braccini 2, 10095 Grugliasco, TO, Italy; ilario.ferrocino@unito.it

**Keywords:** robiola di roccaverano, goat’s milk cheese, mycobiota, high throughput sequencing

## Abstract

Robiola di Roccaverano is a Protected Designation of Origin (PDO) cheese from the Piedmont region of Italy. In this study, the mycobiota occurring during Robiola di Roccaverano production was elucidated. Samples of milk, Natural Milk Cultures (NMC), curd, 5- and 15-days ripened cheese were collected from one dairy plant and the mycobiota was analyzed by the metataxonomic approach. Milk samples showed a high diversity and *Cladosporium*, *Kluyveromyces marxianus*, *Geotrichum candidum* and *Debaryomyces hansenii* were found with higher relative abundance. This mycobiota remains quite stable in NMC and curd matrices although the relative abundance of *K. marxianus* and *G. candidum* yeasts increased significantly and shaped the fungal composition of 5- and 15-day ripened cheese.

## 1. Introduction

Cheese microbiome is complex and highly influenced by the environment and the raw materials used for the manufacturing process. Milk-associated microbiota showed a high biodiversity where Gram-negative (*Pseudomonas*, *Acinetobacter*, *Aeromonas* genera and *Enterobacteriaceae* family) and Gram-positive (*Lactococcus*, *Lactobacillus*, *Streptococcus*, *Staphylococcus*, *Leuconostoc*, *Corynebacterium* and *Macrococcus* genera) are commonly identified [1,2]. However, mould and yeast also called mycobiota are often identified in dairy products where they could play an active role in the developing of the typical organoleptic characteristics of the final products [3]. Mould and yeast species, namely *Debaryomyces hansenii* and *Kluyveromyces marxianus* are involved in sugar fermentation. *D. hansenii* is mainly involved in deacidification of cheese surfaces through lactate utilization ad ammonia production. *D. hansennii* also possesses proteolytic and lipolytic activities and contribute to cheese flavour and texture. Other yeasts, such as *Geotrichum candidum* and *Yarrowia lipolytica*, are of noteworthy interest for the development of flavours and aroma during cheese ripening through the action of lipolytic and proteolytic enzymes [4]. The prevalence of specific yeast species is strictly connected with types of cheese and, in some cases, can be intentionally added to produce several products (e.g., Roquefort, Stilton, Danablue, Camembert, Gorgonzola) [5].

For several decades, the mycobiota inhabiting cheese has been investigated by culture-dependent based methods followed by phenotypic identification combined with PCR-based techniques where the short non-coding ITS regions and the D1/D2 domain of the large subunit ribosomial RNA could be applied [6,7]. Culture-dependent methods are traditionally employed for studying artisanal dairy products, but their performance can be influenced by the complexity of cheese mycobiota. The advent of new molecular tools, such as the high throughput sequencing (HTS) technologies provided a fast and reliable approach to investigate the microorganism occurring in food matrices. These methodologies have been applied in several milk and dairy products for the study of the bacterial communities [8,9,10]; however, mycobiota composition remains less explored. Among HTS, amplification and subsequent sequencing of ITS and 26S rRNA genes are the most common approaches to explore fungal ecology in food matrices [11]. However, metabarcoding do not always allow a sufficient taxonomic resolution for mould identification. It could be susceptible to PCR error or missing details in the reference database used and genomic materials from death cells. A combination of culture-dependent methods with metabarcoding can give complementary insights into the evolution of microorganisms in dairy products.

Furthermore, culture-independent methods can detect rare microbial populations that cannot be identified by culture-dependent methods.

The Piedmont region possesses the highest number of artisanal cheese varieties in Italy where Robiola di Roccaverano recovers a high social and economic interest. This Protected Designation of Origin (PDO) cheese could be obtained from raw milk of Roccaverano and Camosciata Alpina goats breeds and their crosses, from Langhe sheep and from Piemontese and Bruna Alpina cows and their crosses. However, at least 50% of the milk used for the production must be goat’s milk. During manufacturing process, thermization process are not allowed and natural milk cultures (NMC) containing mainly *Lactococcus lactis* are inoculated together with animal rennet for curd formation [12,13,14]. NMC is obtained by back-slopping process where a small amount of the previous days fermented milk is inoculated into fresh milk to start the fermentation process. The curdling procedure takes place in a single container and after 8 h from the beginning, coagulated milk is transferred into a circular mold for draining process. Robiola di Roccaverano can be consumed as fresh cheese, after 5 days of ripening or allowed to ripen for up to 15 days [15].

Several works have been performed for the characterization of the microbiota of Robiola di Roccaverano cheese, however the fungal community involved in this cheese production remains less explored [13,16]. Thus, the present study aimed to understand the variation of the fungal community in the different steps of production of the cheese-making process of Robiola di Roccaverano cheese.

## 2. Materials and Methods

### 2.1. Samples Collection

**Figure 1:**
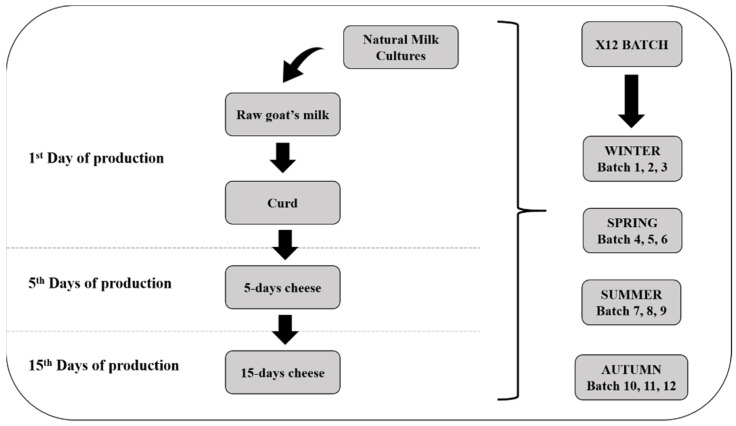
Representative scheme of sampling procedures during the principal production steps of Robiola di Roccaverano cheese.

One artisanal dairy plant of Robiola di Roccaverano cheese has been monitored for one year of production by collecting samples in triplicate in each season (Figure 1). A total of 60 samples was collected from 12 different batches. Three batches from each season were sampled as follows. The first day of production, samples of raw milk, NMC and curd were collected. After 5 and 15 days, fresh and matured cheese above 200 g were collected (Figure 1). Samples were transported in cooled condition in the laboratory and stored at −20 °C until the day of the analysis. Due to the soft texture of Robiola di Roccaverano cheese, samples have been analyzed without distinction among core and rind.

### 2.2. Nucleic Acid Extraction and High-Throughput Sequencing Analysis

For DNA extraction from milk and NMC, 1 mL of samples were firstly centrifuged at 4 °C at 12,000× *g* for 30 min, and supernatant was removed together with fats by using a sterile cotton swab. Pellet was washed with 500 µL of 1X phosphate buffered saline (PBS) and resuspended in 200 µL of ATL buffer (Qiagen, Hilden, Germany) and proteinase K (Qiagen, Hilden, Germany) following the manufacturer’s instruction of the DNeasy Blood and Tissue kit (Qiagen, Hilden, Germany). DNA was then extracted with the DNeasy Blood and Tissue Kit (Qiagen, Hilden, Germany) following the manufacturer’s instruction. For DNA extraction from the cheese, 10 g of sample were homogenized in a sterile stomacher bag with 90 mL of sterile Ringer solution (Oxoid, Milan, Italy) and mixed in a Stomacher 400 Circulator (Seward Ltd, Worthing, UK) at 300 rpm for 3 min. One mL of the first decimal dilution was transferred into 1.5 mL micro-tube and DNA was extracted by using the DNeasy Blood and Tissue kit (Qiagen, Hilden, Germany).

Mycobiota was studied though the amplification of the D1 domain of the 26S rRNA gene using primers and condition described by Mota-Gutierrez et al. [17]. PCR amplicons were purified following the Illumina metagenomic pipeline (Illumina Inc. San Diego, CA, USA). Sequencing was performed with a MiSeq platform (Illumina), generating 250 bp paired-end reads.

### 2.3. Bioinformatic and Statistical Analysis

Paired-end reads were imported in QIIME2 for the bioinformatic analysis [18]. Briefly, primers were removed by cut adapter and sequences were trimmed for low quality, filtered from chimeric sequences and merged by using the dada2 denoise-paired plug in of QIIME2 [19]. Briefly, the quality filtering parameters applied were: [truncLen = c(246,158); trimLeft = c(20,20); maxN = 0; maxEE = c(2,2); truncQ = 3; merged (minimum overlap of 20 bp) and subjected to de-novo chimera removal (per-sample method; 10% of merged sequences detected as chimera and removed). The ASVs table was rarefied at the lowest number of sequences/samples prior to analysis in order to avoid differences due to the sampling depth.

Amplicon sequence variants (ASVs) generated by DADA2 were used for taxonomic assignment using the QIIME2 feature-classifier plugin against an in-house database for the mycobiota [17]. Taxonomic assignment of each ASV was double checked on BLAST suite tools. The ASVs table obtained with QIIME2 displays the higher taxonomy resolution that was reached and when the taxonomy assignment was not able to reach species level, the genus or family name were displayed. QIIME2 diversity script was used to perform alpha and beta diversity analysis.

Anosim statistical test using the VEGAN function of R was used to identify significant differences among different matrices or sampling season. Wilcoxon matched pairs test was used difference in alpha diversity or ASVs abundance as a function of season or sample type. Not-normally distributed variables were presented as median (range interquartile) and box plots represented the interquartile range between the first and the third quartile, with the error bars showing the lowest and the highest value. Bonferroni’s correction for multiple comparisons was applied and *p* value as false discover rate <0.05 or lower was considered as statistically significant.

Sequencing datas were deposited in the Sequence Read Archive of the National Center for Biotechnology Information (NCBI; https://www.ncbi.nlm.nih.gov/ (accessed on 22 June 2021)) under BioProject accession number PRJNA738731.

## 3. Results and Discussion

### 3.1. Alpha and Beta Diversity Analyses

**Figure 2:**
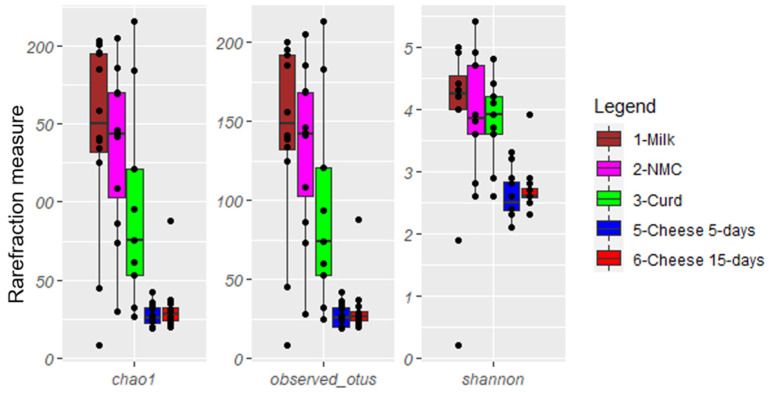
Boxplots describing α-diversity measures of milk, natural milk cultures (NMC), curd, 5- and 15-day ripened cheese.

Rarefaction analysis and the estimated samples coverage for the 26S HTS analysis indicated that there was an adequate coverage for all the samples >99%. The mycobiota composition of Robiola di Roccaverano cheese displayed 22 ASVs at species level, five at genus level and one reaching only class level (Supplementary Table S1). α-Diversity indices (Shannon and Chao1 index) as well as the number of observed species (Figure 2) showed that raw milk, NMC and curd samples displayed a higher level of complexity compared to 5- and 15-day matured cheese (*p* < 0.05).

### 3.2. Taxonomic Diversity of Each Production Steps

**Figure 3:**
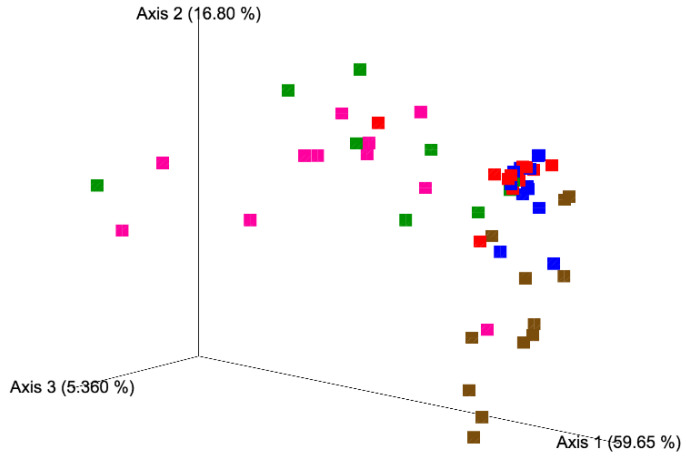
Principal coordinate analysis (PCoA) based on weighted unifrac distance matrix of milk (brown), natural milk cultures (NMC, magenta), curd (green), 5-day ripened cheese (blue), and 15-day ripened cheeses (red).

A different ASVs composition was observed among the five matrices analyzed, in details, PCoA based on Weighted UniFrac distance matrix and the Adonis and Anosim statistical tests (Figure 3) showed a clear separation of samples according to the type of matrices (Supplementary Table S2). However, no differences between season or batch of production were observed.

Although milk samples were highly variable and it was difficult to identify a common fungal composition, there were some ASVs shared among the samples (Figure 3). *Cladosporium* spp. was observed with a relative abundance between 4% and 54% in 10 out 12 samples, where six out 10 samples showed a relative abundance higher than 20%. *K. marxianus* was observed with a relative abundance between 4% and 36% in five out 12 samples and in two samples it reached more than 34%. Instead, in seven samples it was observed with a relative abundance <2%. *G. candidum* displayed a relative abundance between 12% and 63% in 3 out 12 samples, among 1–5% in four out 12 samples, and <1% in four samples. *Cryptococcus* spp. was detected with a relative abundance between 1% and 18% in 10 out 12 samples, while some samples displayed more than 9% of the relative abundance. Finally, two milk samples showed low fungal richness, L2 (batch 1, spring season) and L85 (batch 12, winter season) where *D. hansenii* accounted for the 89,6% and the 97.2% of the relative abundance, respectively. Several studies showed that the taxa mentioned have been commonly associated with goat’s milk [20,21].

The origin of the fungal communities of milk samples could be ascribed to different sources of contamination, which occur during the production chain. Firstly, raw milk could be contaminated from the animal feeding, teat surface of lactating animals, but also by the milking parlor, bulk tank, stable, mode of conservation and operators [1,22,23]. Moreover, mould spores which are frequently dispersed in the air through the air system may occur also in the late stages of the manufacturing process [22]. In this specific case, milk samples have been collected from one single factory, which possess two farms to respect the goat’s dry period. Thus, there are several factors which may indirectly affect the composition of milk mycobiota. Among these sources, feed, watering place, milking parlour and faeces may be colonized by several microorganism and also between these, mould and yeast, which have been transferred to milk during the production [22].

Despite the highest variability being observed in NMC samples, few ASVs were detected with higher relative abundance. *K. marxianus* was the most widespread species: it was found in nine out 12 samples with a relative abundance between 12% and 86%, while in seven samples constituted more than half of the relative abundance. *Cladosporium* genus represents the most important taxa of the NMC, it was observed in NMC with a relative abundance between 13% and 27% in 11 out 12 samples. The strong presence of *K. marxianus* in NMC is not unexpected since this yeast possesses the ability to ferment lactose through β-galactosidase activity [24,25]. *K. marxianus* have been detected as dominant yeast species also in natural whey starter to produce Parmiggiano Reggiano cheese [26,27].

**Figure 4:**
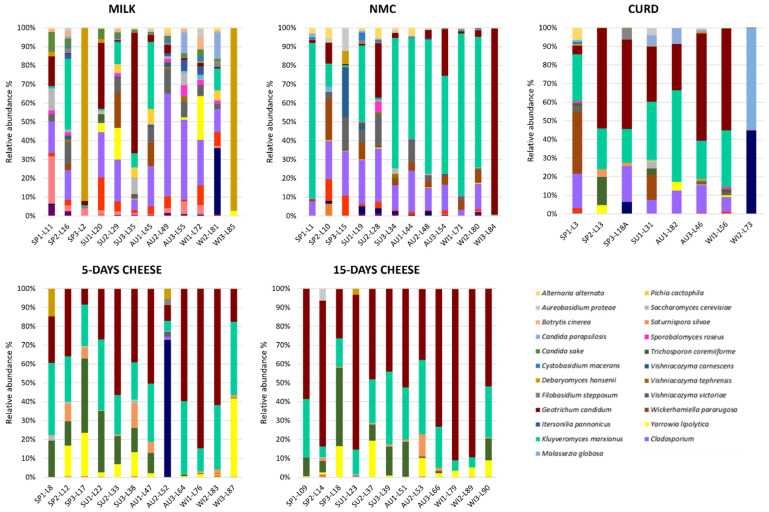
Relative abundance (%) of mycobiota ASVs detected with 26S metataxonomic analysis in milk, natural milk culture (NMC), curd, 5-day and 15-day ripened cheese. Only ASVs with an incidence above 0.5% in at least two samples are shown.

PCoA did not display differences in the fungal composition between 5- and 15-day ripened cheese; however, a common fungal signature was identified (Figure 3). Compared to milk, NMC and curd, cheeses analyzed in this study showed a minor fungal variability. The relative abundance of *K. marxianus* and *G. candidum* together represented more than half of the fungal composition of the 5- and 15-day ripened Robiola di Roccaverano cheese. *K. marxianus* has been isolated from the typical hard-cheese Fiore Sardo, from Pecorino di Farindola as a predominant species [28,29] and from raw milk based French cheeses where the ripening process was influenced through its proteolytic and lipolytic activity [20]. Recently the probiotic potential of *K. marxianus* strains isolated from raw ewe’s milk cheese from Spain have been assessed [30]. *G. candidum* is a common dairy yeast with high resistance to low pH and salt, which could be deliberately added during the cheese production for its relevant technological properties [31]. It is commonly found on the surface of soft smear cheeses and mould-ripened cheese, such as Camembert, but also from several environmental sources like air, water, soil, milk and fruits. This yeast contributes to flavour formation in cheese due to proteolytic and lipolytic enzymes, but is also important for the dairy industry for the control of undesirable microorganisms, such as *Mucor* spp. [32]. *Y. lipolytica* and *Trichosporon coremiiforme* appeared with high relative abundance in the cheese samples, and *Saturnispora silvae*, which was observed rarely in milk and NMC ASVs was detected as sub-dominant species in 11 out 12 samples of Robiola di Roccaverano cheese. The increment of these yeasts in curd and 5- and 15-day ripened cheese may suggest their ability to grow late in the ripening process. *Y. lipolytica* displays a key role for cheese organoleptic characteristics for its strong lipolytic and proteolytic activity during ripening. This yeast, which cannot be deliberately added during the cheese manufacturing process, was poorly detected in milk, NMC and curd but possesses a higher relative abundance in 5 and 15 days of ripening. *Y. lipolytica* was already identified by HTS in French cheese like Tomme d’Orchies and Livarot [33,34]. In addition, one sample of 5-day ripened cheese (L52) displayed a high relative abundance of *Fusarium* genus, which was not detected in curd and 15-day ripened cheese of the same batch. However, it was found in milk and NMC (batch 8, autumn season) (Figure 4). Thus, it could be speculated that the high increment of *Fusarium* spp. may depend on dairy environment contamination due to mould spores, which were dispersed through the air system or even to the contamination of the surfaces where the cheese samples were placed to ripen.

Curd samples were less variable than milk and NMC and *K. marxianus* and *G. candidum* defined the fungal composition of this matrix. A relative abundance among 16% and 55% in all samples was observed for *K. marxianus*. Indeed *G. candidum* was detected with a relative abundance between 24% and 58% in six out of eight samples. In addition, *Cladosporium* was still observed with a relative abundance of 7–18% in six samples out of eight.

The samples analyzed in this study were also matters of a past work already published aimed to characterize the bacterial and fungal diversity of a Robiola di Roccaverano production with culture-dependent techniques [13]. The genera highly detected in the present study, such as *G. candidum*, *Y. lipolytica*, and *K. marxianus* were also among the most abundant strains isolated and identified by RAPD-PCR [13]. Furthermore, few colonies belonging to the species *T. coremiiforme* and *S. silvae* isolated in the past study were detected by HTS. Since there are several further subdominant species detected by sequencing which were not isolated by culturing methods, it is possible that these species were not detectable with the culturing methods applied or with the growth condition chosen. In addition, it might be speculated that species present in high number over competed with the less present ones. HTS and the combination of several methodologies to study food-associated microbiota and mycobiota may allow to obtain more detailed information on the typical microflora of artisanal dairy products.

## 4. Conclusions

In this study, HTS technology was applied to elucidate the mycobiota occurring during Robiola di Roccaverano production as an alternative to traditional culturing methods. Robiola di Roccaverano production possesses a fungal ecosystem quite similar to the ones found in dairy products. Most of the taxa observed in milk and NMC were also observed in curd, 5- and 15-day ripened cheese. The most frequently identified fungal species of Robiola di Roccaverano cheese were constituted by *K. marxianus*, *G. candidum*, *Y. lipolytica*, *T. coremiiforme* and *S. silvae*.

Indeed, it would be of noteworthy interest to perform an in-depth analysis on volatile compound of this PDO cheese in order to correlate the microbiota and mycobiota composition to its peculiar organoleptic characteristics. Several dairy plants need to be considered in order to define the typical core mycobiota of Robiola di Roccaverano.

## Data Availability

Not applicable.

## Supplementary Materials

The following are available online at https://www.mdpi.com/article/10.3390/foods10081859/s1, Table S1: Relative abundance (%) of the taxa detected with 26S sequencing, Table S2: *p*-value and R-square value obtained with Adonis statistical tests.
